# Critical Effects on Akt Signaling in Adult Zebrafish Brain Following Alterations in Light Exposure

**DOI:** 10.3390/cells10030637

**Published:** 2021-03-12

**Authors:** Nicholas S. Moore, Robert A. Mans, Mackenzee K. McCauley, Colton S. Allgood, Keri A. Barksdale

**Affiliations:** Georgia Southern University, Armstrong Campus, Savannah, GA 11935, USA; nmoore3@augusta.edu (N.S.M.); rmans@georgiasouthern.edu (R.A.M.); mm40022@georgiasouthern.edu (M.K.M.); colton.shepherd15@gmail.com (C.S.A.)

**Keywords:** AKT, GSK3β, HSP70, zebrafish, enrichment, light

## Abstract

Evidence from human and animal studies indicate that disrupted light cycles leads to alterations of the sleep state, poor cognition, and the risk of developing neuroinflammatory and generalized health disorders. Zebrafish exhibit a diurnal circadian rhythm and are an increasingly popular model in studies of neurophysiology and neuropathophysiology. Here, we investigate the effect of alterations in light cycle on the adult zebrafish brain: we measured the effect of altered, unpredictable light exposure in adult zebrafish telencephalon, homologous to mammalian hippocampus, and the optic tectum, a significant visual processing center with extensive telencephalon connections. The expression of heat shock protein-70 (HSP70), an important cell stress mediator, was significantly decreased in optic tectum of adult zebrafish brain following four days of altered light exposure. Further, pSer473-Akt (protein kinase B) was significantly reduced in telencephalon following light cycle alteration, and pSer9-GSK3β (glycogen synthase kinase-3β) was significantly reduced in both the telencephalon and optic tectum of light-altered fish. Animals exposed to five minutes of environmental enrichment showed significant increase in pSer473Akt, which was significantly attenuated by four days of altered light exposure. These data show for the first time that unpredictable light exposure alters HSP70 expression and dysregulates Akt-GSK3β signaling in the adult zebrafish brain.

## 1. Introduction

Evidence from animal models and human studies have demonstrated that abruptly altered light cycles, poor sleep, and sleep deprivation exert profoundly negative impacts on brain function and general health. In fact, shift work has been identified as a risk factor for developing cancer [1] and Alzheimer’s disease (AD), and sleep deprivation exacerbates cognitive dysfunction and pathology in a mouse model of AD [2]. Gene expression patterns associated with circadian rhythms have been investigated for decades, as have the mechanisms by which light entrains circadian clocks in various species, but the molecular underpinnings connecting interruptions of circadian functions with cognitive dysfunction and neuropathology are only beginning to be investigated [3].

Akt is an important regulator of cell survival, cell death, mitochondrial function, and learning and memory processes [4,5,6,7]. Akt is regulated by phosphorylation: phosphorylation on the serine-473 or threonine-308 residues via phosphatidylinositol 3-kinase (PI3K) is activating and promotes long term potentiation of synaptic strength, cell survival, microglial phagocytic activity, and increased neurogenesis in the healthy brain in response to enrichment, growth factors, synaptic strengthening, and cell survival signaling [7,8,9]. Akt, in turn, phosphorylates GSK3β on the serine-9 residue, which serves to decrease its activity. This signaling is essential to experience-dependent neuroplasticity [7]. Recent evidence indicates the neuroprotective functions of Akt may be impaired after disruption of the sleep/wake cycle. Specifically, treatment of microglial cells with orexin-A/B—a major modulator of the sleep-wake cycle which mimics sleep deprivation conditions-impaired phagocytosis of amyloid beta by microglia, and this deficit was associated with downregulation of PI3K and Akt [10]. Akt may therefore represent a molecular link between disrupted circadian functions and neuropathology.

GSK3β impacts numerous cellular processes in the brain associated with cell survival [11], inflammation [12], learning, memory [7,13,14] and development [15]. Additionally, the dysregulation of GSK3β has been linked with the cognitive abnormalities and pathologies of AD [16], schizophrenia [17], fragile-X mental retardation [18,19], mood disorders [20,21], ischemic stroke and other excitotoxicity-related disorders [22]. The phosphorylation state of GSK3β regulates its kinase activity—phosphorylation of the serine 9/21 residues by Akt or phospholipase C (PLC) is inhibitory, and this is generally regarded as the pro-cognitive and protective form [23]. Neuroinflammation, a key aspect of AD eitiology and of central importance to injury sustained after ischemic stroke and subarachnoid hemorrhage, is reduced by phosphorylation of GSK3β [22]. In primary neurons, in which glutamate inhibits the Akt/GSK3 pathway and promotes cell death, GSK3 inhibitors protect primary neurons from glutamate-induced cell death [24]. Several other hallmark pathologies of AD—overproduction of soluble amyloid beta oligomers, tau hyperphosphorylation, deficits in synaptic plasticity and cell death—appear to converge upon hyperactive GSK3β, and the therapeutic benefit of inhibiting it. In mice, GSK3β phosphorylation follows a circadian rhythm, and genetically manipulating GSK3β phosphorylation impacts clock gene periodicity in the hippocampus [25]. GSK3β signaling clearly modulates a host of brain functions, including circadian rhythmicity, but the question of whether disruption of circadian function may cause GSK3β dysregulation has not been evaluated. Additionally, a link between acute light exposure and effects on pGSK3B has been identified [26]. It is therefore an open question as to whether altered light cycles, sleep deprivation and other stressors on the circadian system exert adverse effects on brain function by disrupting normal GSK3β signaling.

Heat shock proteins (HSPs) constitute a well-studied family of proteins that confer protection to cells during a host of stressors. Acting as chaperones, HSPs restore aberrantly misfolded proteins [27]. Additionally, HSPs inhibit apoptotic cell death pathways [28,29] and attenuate inflammation [30,31,32,33]. Stressors known to induce the protective functions of HSPs include heat [34], ischemia [35,36], oxidative stress [37,38,39], infections, heavy metals and toxins [27]. Notably, a relationship between HSP70 and circadian functions has been detected—in diurnal mammals, HSP70 levels are known to decline at night and increase during light hours [40,41]. Additionally, sleep deprivation has been shown to stimulate HSP70 expression in the rat cerebral cortex [42] and in several areas of mouse brain [43]. Finally, two days of light interference treatments during dark hours, a stressor and disruptor of normal sleep in diurnal animals, has been shown to stimulate HSP70 in the brain of golden spiny mice [41].

Zebrafish—which have emerged as a powerful animal model for neuroscience-exhibit a diurnal circadian rhythm, a well-characterized sleep-like state, and sleep disruption in response to pharmacology, light exposure, and other protocols used in mammals for the purposes of sleep deprivation [44,45,46,47,48,49,50]. As in mammals, circadian functions of peripheral tissues are affected in a light-sensitive manner by a centralized structure—the pineal gland—which coordinates melatonin release. In striking contrast to mammals, however, light sensitivity is evident in nearly every tissue in zebrafish, imparting the ability for individual cells in peripheral tissues to reset their molecular clocks in direct response to light [51,52,53]. It is known that extended light exposure during the dark phase impairs sleep in zebrafish, and that neurogenesis decreases in the brain of fish after light-induced sleep deprivation [54]. Additionally, gene expression changes in response to altered light exposure have been described [54]. However, detailed investigations into how sleep deprivation and/or altered light may affect intracellular signaling pathways in the zebrafish brain have not been conducted. In the current study, a causal relationship between abnormal patterns of light exposure (a commonly used approach for sleep alteration in many animal models [1,2,41,54]) and alterations in GSK3β, Akt, and HSP70 are examined in the brains of adult zebrafish via exposure to four days of altered, unpredictable light exposure

## 2. Materials and Methods

### 2.1. Zebrafish Husbandry

Adult zebrafish were housed as described by Mans et al., 2019 [55]. All experimental procedures were approved by the Georgia Southern University Institutional Care and Use Committee.

### 2.2. Light Cycle Alteration

**Figure 1:**
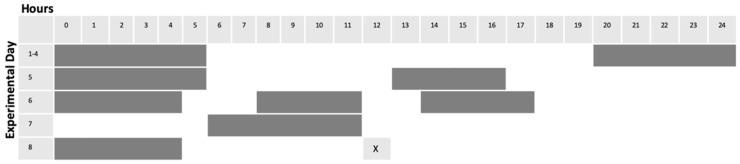
Scheme for alteration of light cycles. Gray bars indicate lights OFF, while white areas indicate lights ON.

Adult zebrafish were subjected to light alteration as follows. Single fish were placed in 2.8 L tanks equipped with carbon filtration (Tetra Whisper 4, Spectra Brands, Blacksburg, VA, USA), and tanks were placed in light-isolated boxes equipped with LED (light emitting diode) lighting (50 C9 White Christmas Lights, Holiday Time, Bentonville, AR, USA) connected to timers (MyTouchSmart Indoor Digital Timer, General Electric, New York, NY, USA). Following a four-day acclimation period in which light cycles were maintained on the standard 14 h on/10 h off protocol (lights ON from 600 h until 2000 h), experimental fish were then subjected to 4 days of unpredictable alterations in light cycles. Control tanks were maintained on standard 14/10 light cycle noted above. Feeding times remained constant for all conditions, and occurred between 1200 h and 1300 h each day (Figure 1). For each replicate (6 replicates for HSP70 expression investigation, 8 replicates for Akt/GSK3β investigation), sampling order was interleaved as an internal control. 2.8 L tanks equipped with carbon filtration (Tetra Whisper 4), and tanks were placed in light-isolated boxes equipped with LED lighting (50 C9 White Christmas Lights) connected to external timers (MyTouchSmart Indoor Digital Timer). Following a four-day acclimation period in which light cycles were maintained on the standard protocol, two boxes maintained the standard light cycle. The third fish was subjected to 4 days of alterations in light exposure (Figure 1). After the 8-day period, the control fish was subject to no enrichment. 5 min of environmental enrichment was carried out for the other two fish: one on normal light cycle (ENR) and one on “altered light” cycle (EN/AL) (protocol developed using Oliviera, 2015 [56]). Enrichment was accomplished by external remote control of LED adhesive light strips (Good Earth Lighting, Plug-In Tape Light with Remote, Black (Mount Prospect, IL)) affixed to the inside of the light isolation boxes, which alternated blue and green on a 5 s interval for five minutes. Following enrichment, fish were anesthetized and prepared for sample collection. For each replication, sampling order was interleaved as an internal control.

### 2.3. Dissection and Sample Collection

Fish were anesthetized using tricaine methanesulfonate (300 μg/mL) until there was no response to tail pinch. The fish were decapitated, and heads were stabilized on foam blocks submerged in ice-cold artificial cerebrospinal fluid (ACSF) consisting of NaCl (120 mM), KCl (3.5 mM), CaCl (2 mM), MgSO_4_ (1.3 mM), MgCl_2_ (1.3 mM), NaH_2_PO_4_ (1.25 mM), NaHCO_3_ (26 mM), and glucose (11 mM). Telencephalon and optic tectum were removed and placed immediately into homogenization buffer containing T-Per (Tissue Protein Extraction Reagent, Fisher Scientific, Pittsburg, PA, USA) containing protease and phosphatase inhibitor cocktails (Roche). Samples were homogenized with a Dremel rotary tool with teflon pestle attachment for 60 s followed by 1-min centrifugation at 1000× *g* and immediate freezing at −20 °C. For sample clarification, thawed samples were centrifuged at 5000× *g* for 10 min and supernatants were removed for protein assay and immunoblotting.

### 2.4. Western Blotting

Protein concentration of homogenates was determined using a NanoDrop spectrophotometer (ThermoFisher, Waltham, MA, USA). In brief, 2 μL of sample was added to the pedestal and absorbance was measured at 280 nm. Protein concentrations were compared to a bovine serum albumin (BSA) standard curve of known concentrations. Total protein concentration was reported in μg/μL units. Samples totaling a 30 μL volume and containing 20 μg of protein were prepared in SDS (sodium dodecyl sulfate) sample buffer (BioRad) using standard sample preparation protocol and as reported by Barksdale, 2009 [57]. Samples were resolved using SDS-PAGE (sodium dodecyl sulfate-polyacrylamide gel electrophoresis) onto 10% polyacrylamide gels of 1.5 mm thickness, then transferred to PVDF (polyvinylidene fluoride) membranes using discontinuous semi-dry transfer (BioRad). Following transfer, membranes were blocked in 5% milk with tris-buffered saline with 0.02% Tween (TBST) for one hour, followed by application of primary antibody (HSP70: GENETEX GTX25442), 1:2000; total-Akt: Cell Signaling (Rabbit mAb #4691) 1:2500; total-GSK3β (Rabbit mAb #5676): Cell Signaling 1:2500) in 2.5% milk/TBST. Primary antibodies to pSer473-Akt (Cell Signaling (Rabbit mAb #4060), 1:5000) and pSer9 GSK3β (Cell Signaling (Ab #9331), 1:5000) were incubated in 2.5% bovine serum albumin (BSA)/TBST [55,58].

Primary antibodies were incubated overnight at 4 °C. Secondary antibodies (HRP-Conjugated goat anti-rabbit; Cell Signaling, 1:1000) were incubated for 1 h in 2.5% milk/TBST followed by chemiluminescent detection with Clarity Western ECL peroxidase substrate (BioRad, Hercules, CA, USA). Blots were imaged using the ChemiDoc MP Imager with Image Lab software v. 5.1 (BioRad). Protein levels were quantified using densitometry of individual bands using ImageJ Freeware (NCBI). Prior to detection of loading control proteins actin (AbCam, 1:2000) or tubulin (Sigma-Aldrich, 1:5000), blots were stripped using a harsh antibody stripping protocol (AbCam), and absence of residual antibody was confirmed via digital imaging.

### 2.5. Data Analysis

**Figure 2:**
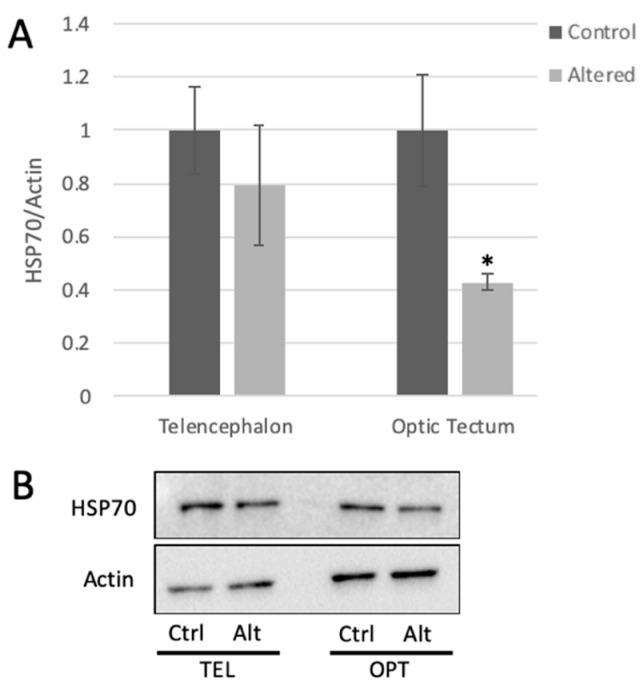
Light cycle alteration significantly decreases HSP70 expression in the optic tectum. (**A**) HSP70 expression measured in the telencephalon and optic tectum from control and light-altered fish. HSP70 protein levels in control samples were set to 1.00. HSP70 levels were normalized to actin. Error bars represent SEM. * *p* = 0.0212, Student’s *t*-test with 95% CI, *t* = 2.7286, *n* = 6. (**B**) Representative Western blot images.

**Figure 3:**
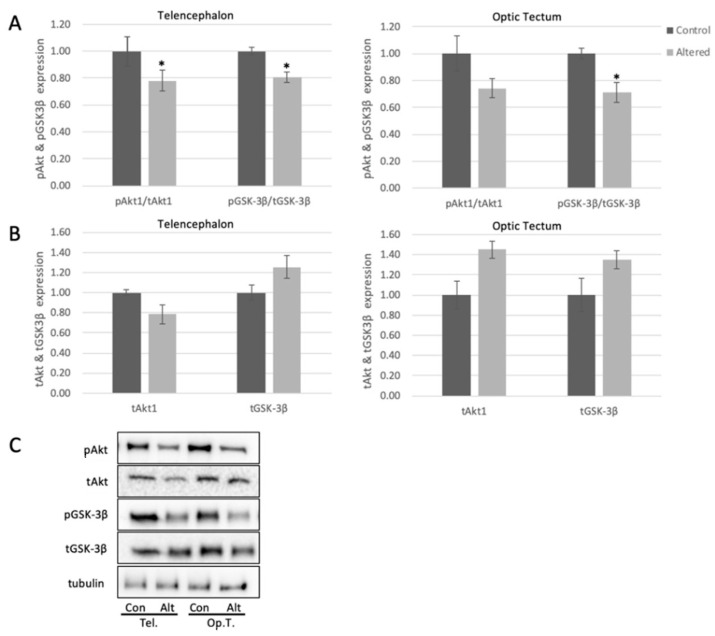
Alterations in light exposure dysregulates Akt and GSK3β in the telencephalon and optic tectum of adult zebrafish. (**A**) Quantified levels of pSer473Akt and pSer9GSK3β in telencephalon (Telencephalon pAKT: *p* = 0.0494, *t* = 2.180; Telencephalon pGSK: *p* = 0.0483, *t* = 2.0754) and optic tectum (Optic Tectum pGSK3β: *p* = 0.0335, *t* = 2.3570) of control and light-altered adult zebrafish. pSer473Akt was normalized to tAkt, and pGSK3β was normalized to tGSK3B. Control protein expression was set to 1.00. (**B**) Quantified levels of Akt and GSK3β in telencephalon and optic tectum of control and light-altered adult zebrafish. Akt and GSK3β were normalized to tubulin. Control protein expression was set to 1.00. Error bars represent SEM. * *p* < 0.05, *n* = 8 (**C**) Representative Western blot images; tubulin shown as loading control.

**Figure 4:**
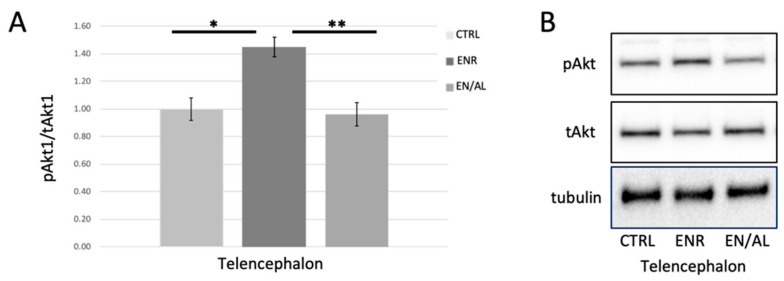
Four days of altered light exposure attenuates enrichment-mediated increase in pSer473Akt. (**A**) Quantified levels of pSer473 measured in telencephalon of control (CTRL), environmentally enriched (ENR), and light altered with enrichment (EN/AL). pSer473Akt protein levels in control samples were set to 1.00, and pAkt levels were normalized to tAkt levels. Error bars represent SEM. (* *p* = 0.00083, Q = 6.60; ** *p* = 0.00339, Q = 0.00339, F = 8.22, 95% CI, *n* = 6) (**B**) Representative Western blot images.

Data were expressed as mean +/− standard error of the mean (SEM). Comparison of data from different treatment groups was performed using Student’s *t*-test (Figure 2 and Figure 3) or ANOVA (Analysis of Variance) (Figure 4 with 95% confidence interval). Protein levels were normalized to control proteins (i.e., pAKT reported as pAkt/tAkt ratio) prior to statistical analysis and graphed with controls set to 1.0.

## 3. Results

### 3.1. HSP70 Expression Is Significantly Diminished Following Light Cycle Alteration

HSP70 protein expression was measured in the telencephalon and the optic tectum using Western blot analysis. HSP70 protein levels showed approximately 50% decrease in the optic tectum of adult zebrafish following four days of light cycle alteration (Figure 2).

### 3.2. Akt and GSK3Β Are Dysregulated Following Exposure to Altered Light Cycles 

pSer473Akt decreased significantly in telencephalon by approximately 20%, and trended to decrease in optic tectum following exposure to alterations in light cycle (Figure 3A), with no significant change to total levels of Akt in the tested brain areas (Figure 3B). These results indicate that the activity of Akt is diminished in the brain following alterations in light exposure, and this may lead to downstream changes to brain biochemistry. To further explore this, we measured the levels of pSer9-GSK3β in the telencephalon and optic tectum. pGSK3β in both regions showed a significant decrease (18% in telencephalon and 30% in optic tectum) with respect to controls (Figure 3A), with no significant change to total levels of GSK3β (Figure 3B).

### 3.3. Light Cycle Alteration Diminishes Enrichment-Related Increase in pSer473Akt

Environmental enrichment has long been reported to increase the activity of Akt, as measured by phosphorylation on Ser473 or Thr308 in rodent and other models [59,60] but has not been tested in adult zebrafish brain. Furthermore, it has been reported in other models that neuroinflammatory processes inhibit enrichment-induced increases in Akt activity [59], and that neuroprotection from stress is accomplished via activation of the PI3K-Akt-GSK3β signaling pathway [59,61,62]. We hypothesized, given the results obtained from light alteration experiments shown in Figure 3, that environmental enrichment would lead to increased pAkt in telencephalon, and that this enrichment-induced increase would be attenuated by unpredictable alterations in light exposure. Figure 4 illustrates that, in telencephalon of adult zebrafish, environmental enrichment caused a robust significant increase in pSer473 Akt levels in telencephalon as expected. This effect was completely blocked by 4 days of exposure to altered light cycle.

## 4. Discussion

Here we have shown significant effects on the biochemistry of adult zebrafish optic tectum and telencephalon in response to four days of unpredictable alterations in light cycles. These effects include a significant decrease in HSP70 expression, significant decrease in Akt activity as measured by pSer473Akt, and significant increase in GSK3β activity as measured by pSer9GSK3β.

Expression of HSP70 decreased significantly in the optic tectum of fish exposed to altered light cycles, but not telencephalon, with respect to controls. These results support previous findings that the optic tectum undergoes significant changes in response to visual experience [63], and further indicate that this may also be true for the adult zebrafish brain. The drop in HSP70 expression in the face light cycle alteration suggests that the adult brain may be less able to cope with external stressors (such as tank sediment, predator threat, or DNA damaging stimuli) when undergoing a potentially stressful stimulus such as unpredictable light exposure. While these data do not match what has been found in rodents specifically, they are substantiated in the results shown in the Yokogawa [46] model of an electric shock stressor, in which cortisol levels rise while HSP70 decreases. Further, changes in the optic tectum may also extend to affect circuitry in the telencephalon, and both regions are important for cognition and experience dependent plasticity [63,64,65].

Akt phosphorylation on the serine-473 residue was significantly diminished in telencephalon of adult zebrafish following four days of light cycle alteration. Interestingly, while not significant, the total levels of Akt showed a trend to increase following exposure to unpredictable light cycles. These data, if investigated further, may solidify that, even though total levels of Akt may increase as part of a compensatory mechanism due to a potential stressor such as sleep alteration, the activity of this pro-survival and pro-plasticity protein remains woefully low. Likewise, GSK3β phosphorylation on the ser-9 residue was significantly decreased in both the optic tectum and telencephalon in zebrafish exposed to altered light cycle, while total levels of GSK3β showed a trend to increase with no significant change. These data suggest presence of increased inflammatory processes, decreased neuronal health, and potential metabolic effects on the sleep-altered brain [66], which could be confirmed upon further investigation. These results, overall, solidify that decreased Akt activity is linked with a significant increase in GSK3β activity in the zebrafish brain, and indicate this pathway is similar to mammalian models. Our results do not rule out other enzymatic effects on Akt or GSK3β activity, but they do confirm presence of this signaling pathway in the adult zebrafish. Since protection of the brain via lithium-induced decrease in GSK3β activity (measured by pSer9GSK3β) is associated with an increase in HSP70 expression in the rodent brain [22], these data may show a similar signaling pathway in the zebrafish brain, in which decreased HSP70 expression occurs concurrently with decreased Akt activity and increased GSK3β activity.

In addition to effects on baseline HSP70 expression, Akt activity and GSK3β regulation, enrichment-related increase in pSer473 was completely attenuated by four days of alterations in light exposure. These results are significant because environmental enrichment had not previously been explored in the context of light exposure or circadian rhythm using zebrafish as a model. These results support many previous reports in multiple mammalian models (human, rat, mouse, monkey) that disruption of normal light exposure alters critical biochemical signaling that is required for homeostasis, cell survival, mood, and experience dependent plasticity [46,67,68,69].

Taken together, our results indicate that the Akt-GSK3β signaling pathway is dysregulated in the telencephalon and optic tectum, accompanied by decreased expression of HSP70 in the optic tectum of adult zebrafish upon light cycle disruption. These results serve to substantiate the future use of adult zebrafish in the investigation of stress-induced pathways and indicate underlying cellular signaling related to learning and memory that occurs in the adult zebrafish brain following as a result of alterations in light cycle. As the Akt-GSK3β signaling pathway is strongly linked to long term potentiation, depression and neuroinflammation, these experiments may illustrate potential biochemical underpinnings of cognitive changes that occur with alterations in light exposure.

## Data Availability

Data are available upon request to the corresponding author.
